# Early ultrasonographic evaluation of idiopathic clubfeet treated with manipulations, casts, and Botox^®^: a double-blind randomized control trial

**DOI:** 10.1007/s11832-015-0633-4

**Published:** 2015-01-22

**Authors:** Alyssa M. Howren, Douglas H. Jamieson, Christine M. Alvarez

**Affiliations:** 1 Department of Orthopaedics, British Columbia’s Children’s Hospital, 1D18-4480 Oak Street, Vancouver, BC V6H 3V4 Canada; 2 Department of Radiology, British Columbia’s Children’s Hospital, Vancouver, Canada; 3 Faculty of Medicine, UBC, Vancouver, Canada; 4 Department of Orthopaedics, Faculty of Medicine, University of British Columbia, Vancouver, Canada

**Keywords:** Clubfoot, Ultrasound, Achilles tendon, Gastrocsoleus, Botox, Onabotulinumtoxin A

## Abstract

**Background:**

The manipulations, casts, and Botox^®^ method for treating idiopathic clubfoot is an alternative non-surgical treatment method. Botox^®^-induced reversible muscle paralysis of the gastrocsoleus enables a physician to manipulate and cast the clubfoot in greater dorsiflexion. Ultrasound is incorporated during the early treatment stages to monitor the underlying physiology of the muscle–tendon unit following Botox^®^.

**Methods:**

Ultrasonographic evaluation was performed parallel to a double-blind randomized control trial administering Botox^®^ or placebo to correct clubfoot. Patients underwent two-dimensional ultrasound to monitor the length changes to the gastrocsoleus and Achilles tendon unit at two time points: pre-injection (baseline) and 6 weeks post-blinded injection. Gastrocsoleus and Achilles tendon length measurements were analyzed among placebo, Botox^®^ and contralateral controls using repeated measures ANOVA.

**Results:**

The baseline gastrocsoleus length of the clubfoot (322.4 pixels) before blinded injection appears shorter than controls (337.5 pixels), but fails to reach significance (*p* = 0.05). The complex length within each of the three treatment groups displayed no significant change between baseline and 6 weeks. The complex–tendon ratio and muscle–tendon ratio of the Botox^®^ treatment group was significantly decreased compared to controls (*p* = 0.049 and 0.042, respectively). Briefly, when expressed as a proportion, an increase in Achilles tendon length and decrease in gastrocsoleus is observed when clubfeet are treated with Botox^®^.

**Conclusions:**

Only in the Botox^®^ treatment cohort did the muscle shrink to uncover tendon (seen as a decreased complex–tendon ratio and muscle–tendon ratio) over the 6-week interval to effectively increase tendon length with respect to the unit as a whole.

**Electronic supplementary material:**

The online version of this article (doi:10.1007/s11832-015-0633-4) contains supplementary material, which is available to authorized users.

## Introduction

Various techniques for the treatment of clubfoot have been utilized, with a current focus on non-surgical techniques and their associated advantages [1]. The Ponseti method has emerged as one of the most commonly used methods. Its initial treatment phase utilizes serial manipulations and casts and typically requires an Achilles tenotomy for correction of the equinus deformity [2]. Ultrasound analysis with a minimum 1-year follow-up post-tenotomy reveal an Achilles tendon with unorganized fibre structure and isolated thickening in the sectioned region [3, 4].

In 2000, an alternative approach to correcting clubfoot was developed, which implements serial manipulations and casts with the adjuvant therapy of onabotulinumtoxin A (Botox^®^), referred to as the MCB method [5, 6]. Botox^®^ allows for the correction of hindfoot equinus, without disrupting the integral muscle–tendon unit.

Injection of Botox^®^ into skeletal muscle initiates a signal cascade, which leads to temporary muscle paralysis of the targeted muscle complex. Before Botox^®^ is endocytosed at the pre-synaptic nerve terminal it is cleaved into a light chain and a heavy chain. The heavy chain allows for selective uptake into the pre-synaptic nerve terminal at the neuromuscular junction. Within the nerve terminal the light chain’s protease activity is activated by the acidic environment, which then permits the light chain to cleave SNARE proteins, inhibiting the release of synaptic vesicles containing the neurotransmitter, acetylcholine [7]. New nerve sprouts extend into the affected area and produce synaptic activity by approximately 50 days. At 3 months post-injection the new nerve sprouts regress and activity of the original nerve terminals is rejuvenated [8].

The physiological and biomechanical changes induced by Botox^®^ on the skeletal muscle complex [9–12] and the accompanying tendon [13–15] have been studied in animal models. Previous research has utilized ultrasound to capture the changes to human muscle architecture post-botulinum toxin-A injection in adult stroke patients [16]. However, to the best of our knowledge there is no literature investigating the effect that Botox^®^ elicits on the structure of the human postnatal muscle–tendon unit using ultrasound.

To investigate the effect of Botox^®^ therapy on the gastrocsoleus and Achilles tendon unit of idiopathic clubfoot, we conducted a two-dimensional (2-D) ultrasound study parallel to a double-blind randomized control trial, thereby providing the opportunity to monitor the response of the muscle–tendon unit to manipulations, casts, and Botox^®^ in vivo.

## Materials and methods

### Study participants

**Fig. 1:**
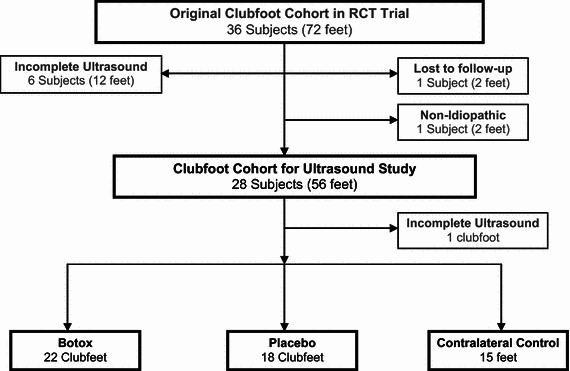
Overview of subjects in randomized control trial (*RCT*) ultrasound study

A total of 36 clubfoot participants were enrolled in the double-blind randomized control trial at BC Children’s Hospital (BCCH) between March 2006 and January 2011. Twenty-eight patients were included in the ultrasound study (Fig. 1). Contralateral feet of subjects diagnosed with a unilateral clubfoot were examined by the orthopaedic surgeon (C.A.) and served as controls.

### Treatment protocol

Upon presentation to BCCH Clubfoot Clinic, clubfoot subjects are treated according to the manipulation and cast, injection, and bracing techniques thoroughly described in previous work [6]. Dimeglio [17] and Pirani [18] scores for each clubfoot are measured at the initial visit. The moment during the manipulation and casting phase at which the forefoot can be abducted beyond 60° but hindfoot equinus persists is referred to as hindfoot stall. Further intervention is required to attain full correction and is therefore followed by a blinded injection of either botulinum toxin type-A (Botox^®^; Allergan, Inc.) or placebo (unpreserved saline). Randomization was by patient and determined by a computer-generated randomization table. An unblinded pharmacist prepared the Botox^®^ and placebo syringes. One orthopaedic surgeon (C.A.) administered every blinded injection. The quantity of Botox^®^ or placebo administered is determined according to the patient’s weight, at a dose of 10 U/kg or 0.1 ml/kg, respectively. The site of injection, as described previously, is the gastrocsoleus muscle belly [6]. For subjects with bilateral clubfeet, the total injection dose is divided equally between the two limbs. All clubfeet were then casted post-blinded injection until full correction was achieved. Correction was then maintained by full-time use of the Dennis Browne bar and corrective shoes. At 6 weeks post-blinded injection a handheld goniometer was used to measure dorsiflexion in flexion (DFF) and dorsiflexion in extension (DFE) for the affected foot.

The ultrasound study focused on two time points: baseline and 6 weeks post-blinded treatment. Baseline was defined as the moment at which the clubfoot reaches hindfoot stall.

### Data collection

The ultrasound evaluation was performed in the radiology department using an Acuson Sequoia 512 ultrasound unit (Siemens Medical) with a high-resolution linear 15L8W probe. Static images and perspective extended field of view images using Siescape technology were obtained.

**Fig. 2:**
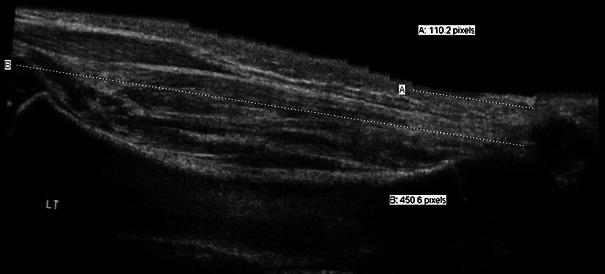
Ultrasound image depicting measurements of (**a**) Achilles tendon length and (**b**) gastrocsoleus muscle and Achilles tendon complex length

The infant was placed prone with knee straight and the foot protruding off the edge of the bed so that it could be readily grasped and dorsiflexed, attempting to achieve 90° between the tibia and foot plantar surface and create a uniform measuring situation. The fixed point for Achilles tendon insertion was the posterior superior corner of the calcaneous bone. The chosen surrogate for origin of gastrocsoleus muscle was the superior lateral margin of the tibia. In Siescape mode, a probe runs smoothly between these two points to produce an extended field of view image. Three suitable images per limb were obtained. Two paediatric radiologists performed the study, one of whom (D.J.) did all the post-imaging calculations. The extended field of view images are composite images providing pixel distances, not direct millimeter measurement. Thus a ratio of “lateral superior tibia” to “posterior superior calcaneous” (representing the muscle tendon complex) divided by the “posterior superior calcaneous” to “Achilles aponeurosis” (representing the upper limit of the Achilles tendon) was calculated (Fig. 2). To account for the potential error associated with the difficultly in precisely delineating the aponeurosis, both measurements were obtained three times per limb. An average value for each measure was calculated for analysis.

### Statistical analysis

Measurements collected at baseline were evaluated between two groups: clubfoot and control. Complex, muscle, tendon, complex–tendon (CT) ratio, and muscle–tendon (MT) ratio measurements for the two groups were compared using a one-tailed *t*-test with equal variance assumed.

Complex, muscle, tendon, CT ratio, and MT ratio measurements for Botox^®^, placebo, and control groups at baseline and 6 weeks post-blinded injection were compared using repeated measures ANOVA (IBM SPSS Statistics, Version 21).

## Results

The distribution of demographics, initial severity scores, and range of motion for clubfoot subjects in the study are presented in Table 1. No adverse drug events occurred during the course of the study.

**Table 1:** Study participant demographics and measurements

Total subjects	28
**Gender**	
Female	6 (21 %)
Male	22 (79 %)
**Average age (months)**	
Baseline	1.44 ± 0.44
6 weeks post-treatment	2.83 ± 0.40
**Average clubfoot severity scores** ^a^	
Pirani	
Botox^®^	5.3
Placebo	5.4
Dimeglio^b^	
Botox^®^	16.6
Placebo	16.5
**Range of motion (6 weeks post-treatment)**	
DFF	
Botox^®^	27.6°
Placebo	24.9°
DFE	
Botox^®^	22.1°
Placebo	19.7°

^a^Measurements collected at first visit to BC Children’s Hospital clubfoot clinic

^b^Dimeglio scores based on 31 clubfeet

Baseline data comparing pre-injected clubfeet to controls (Table 2) characterize a clubfoot with an increased Achilles tendon length, decreased complex length, and decreased gastrocsoleus muscle length. These differences did not, however, reach statistical significance.

**Table 2:** Mean baseline ultrasound length measurements of clubfoot and control muscle–tendon complexes

	Complex	Tendon	Muscle	Muscle–tendon ratio	Complex–tendon ratio
Clubfoot (*n* = 40)	454.6	132.2	322.4	2.49	3.50
Control (*n* = 15)	464.6	127.1	337.5	2.72	3.72
*P* value^a^	0.14	0.18	0.05	0.07	0.07

All measurements recorded in pixel units

^a^One-tailed *t* test between clubfoot and controls at baseline

The average lengths and ratios obtained from the gastrocsoleus and Achilles tendon unit for each treatment group at both time intervals are summarized in Table 3.

**Table 3:** Mean values and standard deviations at baseline (*BL*) and 6 weeks post-treatment (*6W*)

	Complex		Tendon		Muscle		Muscle–tendon ratio		Complex–tendon ratio	
	BL	6W	BL	6W	BL	6W	BL	6W	BL	6W
Control	464.6 ± 24.2	475.0 ± 28.8	127.1 ± 17.4	131.6 ± 17.6	337.5 ± 25.2	343.3 ± 20.9	2.72 ± 0.56	2.65 ± 0.37	3.72 ± 0.56	3.66 ± 0.37
Placebo	451.3 ± 32.6	454.2 ± 27.2	129.3 ± 20.8	136.3 ± 15.1	322.0 ± 29.4	317.9 ± 22.8	2.56 ± 0.52	2.36 ± 0.30	3.57 ± 0.53	3.36 ± 0.31
Botox^®^	457.3 ± 33.4	454.4 ± 22.8	134.6 ± 16.1	141.5 ± 24.4	322.6 ± 33.8	312.9 ± 26.2	2.44 ± 0.45	2.30 ± 0.58	3.44 ± 0.46	3.32 ± 0.59

All measurements recorded in pixel units

Repeated measures ANOVA found no significant difference within each of the three treatment groups with respect to baseline and 6-week time measurements for the complex, tendon, and muscle lengths (Online Resource 1).

Analysis of baseline and 6 weeks post-length measurements between the three treatment groups was assessed among five parameters: complex (*F*_2,52_ = 2.500, *p* = 0.092); tendon (*F*_2,52_ = 1.732, *p* = 0.187); muscle (*F*_2,52_ = 4.919, *p* = 0.011); CT ratio (*F*_2,52_ = 3.131, *p* = 0.052); and MT ratio (*F*_2,52_ = 3.294, p = 0.045). Accompanying pairwise comparisons between Botox^®^ or placebo groups and control are summarized in Table 4. Of heightened importance is the MT ratio (*p* = 0.042) and CT ratio (*p* = 0.049) of the Botox^®^ group exhibiting a significant change over time compared to controls, whilst the placebo ratios displayed no difference from controls (MT ratio *p* = 0.265, CT ratio *p* = 0.288).

**Table 4:** Pairwise comparisons between placebo, Botox^®^, and control

	Pairwise comparison		Mean difference^a^	Standard error	*p* Value
Complex	Control	Placebo	−17.009	8.053	0.118
		Botox^®^	−13.933	7.713	0.230
Tendon	Control	Placebo	3.432	5.007	1.000
		Botox^®^	8.693	4.796	0.227
Muscle	Control	Placebo	−20.442	8.005	0.041*
		Botox^®^	−22.627	7.667	0.014*
Muscle–tendon ratio	Control	Placebo	−0.226	0.130	0.265
		Botox^®^	−0.316	0.124	0.042*
Complex–tendon ratio	Control	Placebo	−0.224	0.132	0.288
		Botox^®^	−0.314	0.127	0.049*

All measurements recorded in pixel units

^a^Negative value indicates the placebo or Botox^®^ measurement is less than control

*Statistically significant

The pairwise comparisons revealed no significant difference between the placebo and Botox^®^ groups with respect to tendon (*p* = 0.759), muscle (*p* = 1.000), and complex (*p* = 1.000) length. Similarly, the CT ratio (*p* = 1.000) and MT ratio (*p* = 1.000) showed no significant difference between placebo and Botox^®^ treatment.

Repeated measures ANOVA found no significant interaction, indicating that the three treatment groups behaved similarly over the two time points (Online Resource 2).

To summarize the results: (1) no significant lengthening of the gastrocsoleus–tendon complex for the three groups was observed over a 6-week interval, (2) a certain amount of tendon creep is seen with casting alone, and (3) only the Botox^®^ group showed increased tendon length and decreased muscle length when expressed as a proportion.

## Discussion

Incorporation of 2-D ultrasound in the double-blind randomized control trial investigating Botox^®^ versus placebo for clubfoot correction enabled our team to monitor the underlying physiological response to the treatment. The main results describe the clubfoot cohort’s Achilles tendon and gastrocsoleus lengths before blinded intervention, and identify the underlying changes to the muscle–tendon unit at 6 weeks post-blinded treatment. Ultrasound evaluation of the Achilles tendon after exposure to Botox^®^ depicts a significant decrease in MT ratio and CT ratio over the 6-week time frame.

After a thorough review, we found limited literature that used ultrasound to describe the gastrocsoleus and Achilles tendon complex lengths of an untreated clubfoot [19]. Our findings show that the post-natal clubfoot appears to have a shorter gastrocsoleus muscle length; this value, however, falls short of reaching significance. Ultrasound measurements of the gastrocsoleus and Achilles tendon of subjects with cerebral palsy (CP) present findings similar to our clubfoot study. Children with spastic CP have a shorter gastrocsoleus muscle belly length than normally developed children [20–22]. A decrease in fascicle length is noted not to be the underlying cause for this discrepancy [21, 23]. Furthermore, children with spastic CP and equinus gait have longer Achilles tendons than controls [22]. While our baseline analysis of the clubfoot muscle–tendon unit characterizes a longer Achilles tendon, this value does not reach significance. It is postulated for the CP population that in order to accommodate for the shortened gastrocsoleus, the increased Achilles tendon length enables the muscle–tendon unit to extend through a suitable range of motion [24].

Tendons are the principal elastic component of the muscle–tendon complex, harnessing the ability to transform energy and transmit force [25–27]. Ultrasound studies examining the Achilles tendon of children with clubfoot pre- and post-tenotomy reveal a successful continuous tendon approximately 3–6 weeks post-operatively [3, 4, 28, 29]. However, long-term ultrasound studies show persistent scar thickening of the cut tendon and irregularities of the tendon fibres [3, 4]. These ultrasound findings are presumably related to gait analysis results post-tenotomy, describing clubfoot subjects with a decrease in ankle power generation of 17 % [30].

The goal of an Achilles tenotomy in the setting of clubfoot is to lengthen the tendon and thereby the muscle–tendon unit, which allows for increased dorsiflexion of the ankle. A typically developed tendon is predominantly comprised of collagen type I [31]. During the healing phases of a ruptured Achilles tendon, tenocytes fill the gap with a significantly increased amount of collagen type III fibres [32]. Collagen type III fibres are thinner and as a result have a decreased ability to withstand tensile forces [33]. This divergence from the natural microanatomy of a tendon likely reduces the viscoelastic properties of the muscle tendon unit and alters the effective lever arm, resulting in decreased power generation. Botox^®^ presumably does not modify the elastic modulus or the architecture of the integral Achilles tendon. Additionally, a tenotomy causes measurable recoil in the associated muscle complex, leading to shortening of that particular muscle [34].

Tendons have a number of thoroughly established visoelastic properties, one of which is the model termed tendon creep. This visoelastic property states that when a tendon is held under constant tension a noticeable increase in tendon length is observed. This phenomenon can be seen when an individual is treated with corrective braces or casts, and highlights the ability of a tendon to respond to an external stress by increasing its strain, allowing for biomechanical equilibrium to be re-established [25, 35]. Tendon creep is a potential explanation for the slight lengthening of the Achilles tendon observed in our placebo and Botox^®^ treatment groups, both of which received manipulations and casts. Importantly, once the desired range of motion is achieved, the creep needs to be continually preserved through a maintenance program.

The model of Achilles tendon creep perhaps initially seems counterintuitive with respect to the mechanism of Botox^®^. However, Botox^®^ causes reversible muscle paralysis of the gastrocsoleus, which removes the antagonistic contraction of the shortened muscle against the Achilles tendon. This enables the clubfoot to be manipulated and held through an extended range of dorsiflexion, which is sustained by casts, and the resultant tensile force is applied unobstructedly on the Achilles tendon. Therefore, the applied stretch is increased and consequently facilitates creep of the Achilles tendon.

A decrease in both the MT ratio and CT ratio following Botox^®^ intervention indicates an underlying change in the composition of the muscle–tendon unit. The placebo group behaves in a similar manner with respect to Achilles tendon and gastrocsoleus lengths over time, but the ratios are not significantly different from controls. The larger decrease in muscle length for the Botox^®^ group is potentially explained by the knowledge that the slack sarcomere length, muscle mass, and fibre cross-sectional area of muscle (normal rat model) treated with Botox^®^ is decreased compared to untreated controls [12]. The shortened gastrocsoleus length seen in neutral position, however, does not seem to influence the clinical range of motion of the clubfoot (DFF = 27.6°, DFE = 22.1°). Alternatively, if a muscle injected with Botox^®^ is analyzed for maximal length, an increase in length is observed [36]. Future studies of this nature should include ultrasound imaging of the foot in varying degrees of flexion and extension. This would permit added insight into the effect of Botox^®^ on the complex and allow for investigation of muscle and tendon length relationships with range of motion parameters.

There are a few limitations to this study that warrant consideration when interpreting the results. Foot pressure analysis characterizes the unaffected contralateral foot as having significantly different plantar pressure profiles compared to the feet of normally developed individuals [37, 38]. These findings do not indicate a direct irregularity in the underlying anatomy of the contralateral clubfoot, but should be taken into consideration, as our control group consisted solely of contralateral feet.

Further consideration should also be made when extending the findings of this study to postulate the effects Botox^®^ will have on a typically developed muscle and tendon, as histological investigation into the pathology of the affected clubfoot muscle has produced conflicting results [39–41].

To gain further insight into the physiological effect of Botox^®^ on the injected gastrocsoleus and Achilles tendon unit, future clinical research should implement a validated 3-D ultrasound technique [42, 43].

Injection of Botox^®^ into the gastrocsoleus of a clubfoot with persistent hindfoot equinus enables redistribution of the muscle–tendon unit. Botox^®^-induced muscle paralysis allowed for increased Achilles tendon length at the expense of muscle. Consideration should be directed towards the benefits of a long duration of non-tensile muscle and the effective pain management that Botox^®^ provides.

## Electronic supplementary material

Below is the link to the electronic supplementary material.

Supplementary material 1 (DOC 31 kb)

Supplementary material 2 (DOC 30 kb)
