# Supplementary material for: Early ultrasonographic evaluation of idiopathic clubfeet treated with manipulations, casts, and Botox®: a double-blind randomized control trial
Source: J Child Orthop. 2015 Jan 22;9(1):85–91. doi: 10.1007/s11832-015-0633-4 (PMC4340848; doi:10.1007/s11832-015-0633-4)
Supplement: Supplementary file 2 — Supplementary material 2 (DOC 30 kb) [file 11832_2015_633_MOESM2_ESM.doc]

**Online Resource 2**

**Title:** Early Ultrasonographic Evaluation of Idiopathic Clubfeet Treated with Manipulations, Casts, and Botox®: A Double-Blind Randomized Control Trial

**Journal:** Journal of Children’s Orthopaedics

**Authors:**

^a^Alyssa M. Howren, BSc;

^bc^Douglas H. Jamieson, MD, FRCPC;

^acd^Christine M. Alvarez, MD, FRCSC, MSc

**Author Affiliations:**

^a^Department of Orthopaedics, British Columbia’s Children’s Hospital

^b^Department of Radiology, British Columbia’s Children’s Hospital

^c^Clinical Associate Professor, UBC, Faculty of Medicine

^d^Department of Orthopaedics, Faculty of Medicine, University of British Columbia

**Correspondence**

Alyssa M. Howren

E-mail: [ahowren@cw.bc.ca](mailto:ahowren@cw.bc.ca)

**Online Resource 2.** Analysis of variance output for interaction of treatment and time

|  | **F ratio** | **Significance** |
| --- | --- | --- |
| **Complex** | 0.693 | 0.505 |
| **Tendon** | 0.050 | 0.951 |
| **Muscle** | 1.253 | 0.294 |
| **Complex-Tendon Ratio** | 0.227 | 0.798 |
| **Muscle-Tendon Ratio** | 0.192 | 0.826 |
